# Genome Sequencing and Analysis of the Filamentous Fungus *Penicillium sclerotiorum* 113, Isolated after Hurricane Sandy

**DOI:** 10.1128/genomeA.01153-16

**Published:** 2016-11-23

**Authors:** Guohua Yin, Yuliang Zhang, Kayla K. Pennerman, Sui Sheng T. Hua, Qixing Huang, Anping Guo, Zhixin Liu, Joan W. Bennett

**Affiliations:** a Department of Plant Biology and Pathology, Rutgers, The State University of New Jersey, New Brunswick, New Jersey, USA; b Key Laboratory of Biology and Genetic Resources of Tropical Crops, Ministry of Agriculture, Institute of Tropical Bioscience and Biotechnology, Chinese Academy of Tropical Agricultural Sciences, Haikou, Hainan, China; c U.S. Department of Agriculture, Western Regional Research Center, ARS, Albany, California, USA

## Abstract

*Penicillium sclerotiorum* is a distinctive species within the genus *Penicillium* that usually produces vivid orange to red colonies, sometimes with colorful sclerotia. Here, we report the first draft genome sequence of *P. sclerotiorum* strain 113, isolated in 2013 in the aftermath of Hurricane Sandy from a flooded home in New Jersey.

## GENOME ANNOUNCEMENT

*Penicillium sclerotiorum* was first isolated from an air sample in Java, Indonesia, and described in 1937 (1). It has subsequently been isolated from Africa, Asia, and North America (2, 3). The fungus usually produces distinctive bright orange pigments, some of which have been identified as carotenoids (4). It also makes several xylanases (5, 6), as well as sclerotiorin, a secondary metabolite that has been used in anti-acne creams and as an inhibitor of aldose reductase (7–10). Furthermore, it has biotechnological potential for producing calcium malate from glucose (11). A recent report showed that *P. sclerotiorum* is the etiological agent of postharvest fungal diseases of pomegranate in Spain (12). Here, we report and characterize the genome of *P. sclerotiorum* 113, a strain isolated from a home in Manasquan, New Jersey, that was flooded with marine water in 2013 during Hurricane Sandy (13).

For genomic sequencing, we grew the fungus in potato dextrose broth with shaking at 200 rpm, 25°C for 7 days. We used an OMEGA Bio-Tek E.Z.N.A fungal DNA midi kit to extract genomic DNA and prepared 500-bp, 2-kb, and 8-kb fragments using an Illumina MiSeq benchtop sequencer. The sequence depths for each library were 186×, 94×, and 67×, respectively. Genome assembly using SOAPdenovo version 2.04 (http://soap.genomics.org.cn) resulted in 737 contigs, with an *N*_50_ value of 157,787 bp and a G+C content of 48.63%. We estimated the genome size of *P. sclerotiorum* to be about 35 Mb based on a 17 *k*-mer statistical analysis.

The assembled genome was annotated using the MAKER2 program (14). *P. sclerotiorum* 113 has an estimated 12,649 putative genes averaging 1,418 bp in length and comprising 52.7% of the whole genome. Repetitive sequences were 279,139 bp in total, constituting 0.82% of the genome. We compared the orthologous genes shared by *P. sclerotiorum* and five other *Penicillium* strains available in public databases (*P. griseofulvum* PG3, *P. expansum* R19, *P. expansum* ATCC 24692, *P. solitum* RS1, and *P. glabrum* DAOM239074) using OrthoMCL. A total of 6,249 common orthologous genes were identified. *P. sclerotiorum* 113 contained the highest number of nonorthologous genes (2,205), followed by *P. glabrum* DAOM239074 (1,960), *P. expansum* ATCC 24692 (866), *P. solitum* RS1 (672), *P. griseofulvum* PG3 (509), and *P. expansum* R19 (326). This is the first report of the genome of *P. sclerotiorum* and its analysis. These data will be a foundation for improving our understanding of the distinctness of *P. sclerotiorum* from other members of the genus, its cosmopolitan distribution, and its biotechnological potential.

### Accession number(s).

The whole-genome sequence of *P. sclerotiorum* 113 has been deposited at DDBJ/ENA/GenBank under the accession number MJCA00000000. The version described in this paper is the first version, MJCA01000000.
